# Recent Work on Leukæmia and Some Allied Affections

**Published:** 1908-12

**Authors:** J. Michell Clarke


THE BRISTOL

MEDICO-CHIRURGICAL JOURNAL.

*"Scire est nescire, nisi id me
Scire alius sciret."*

DECEMBER, 1908.

### RECENT WORK ON LEUKÆMIA AND SOME ALLIED AFFECTIONS.

The President's Address, delivered on October 14th, 1908, at the opening of the Thirty-fifth Session of the Bristol Medico-Chirurgical Society.

BY

J. MICHELL CLARKE, M.A., M.D.Cantab., F.R.C.P.

My first and most pleasing duty is to return my grateful thanks to the members of this Society for the honour they have conferred upon me by electing me their President for the year. It is an honour which I appreciate very highly, and more especially because my father occupied this chair before me just upon thirty years ago. Under such circumstances, it is inevitable that one's thoughts should go backwards for a generation, and consider the changes that have taken place in the art and practice of medicine during that period. So that in searching for a suitable subject for this address, it naturally occurred to me to select a group of diseases in which medicine has greatly advanced during recent years, about which comparatively little was known thirty years ago, and also one of which I have had sufficient personal experience.

I intend, therefore, to take the subject of leukæmia, and one or two affections allied to it. Great advances have been made in our knowledge of these diseases in the last few years, obscure as in many respects they still are.

I may be permitted to give a brief summary of the history of leukæmia. Virchow first clearly recognised the characteristic features of the disease in 1845, although just before him J. Reid and Hughes Bennett had described a condition *post mortem* which, according to the latter, was fatal "from suppuration of the blood."[1] According to the association of the blood-change either with an enlarged spleen or enlarged lymphatic glands, leukæmia was divided into (1) the splenic, and (2) the lymphatic varieties. The next step was made by the important discovery by Neumann of the origin of the leucocytes in the bone-marrow. He also held the view, which is becoming generally accepted at the present day, that the bone-marrow is affected in all cases of leukæmia. As a result of his discovery and its immediate bearings on leukæmia, to the original varieties were added two more, (3) the spleno-medullary, and (4) the myelogenic forms. A further step in advance was due to the introduction of staining methods for blood films, and thereby the differentiation of the various forms of leucocyte, by Ehrlich.

The diagnosis of the leukæmic blood-condition was originally based on the numerical increase of the white cells and their proportion to the reds. The more thorough examination of the blood in all kinds of diseases led to the discovery of the occurrence of leucocytosis under various conditions, and this produced fresh difficulty, for on the basis only of numerical increase of leucocytes it is impossible to separate the higher grades of leucocytosis from leukæmia, especially from those cases of leukæmia in which the actual number of leucocytes is not greatly increased. By Ehrlich's work, however, the diagnosis of leukæmia was established on the characters of the cells present, and the characteristic features of the leukæmic blood-picture demonstrated. He distinguished two distinct forms of the disease, characterised by the kind of cell predominating in the blood. The old distinction of the splenic and lymphatic forms, based on the changes found in the organs clinically and *post mortem*, gave way to one based on the characters of the blood-picture solely, and hence myeloid and lymphatic leukæmia were differentiated. From the views of Ehrlich as to the production of myelocytes, and generally of cells with granules in their protoplasm by the marrow, and of lymphocytes or non-granular cells generally by the lymphatic glands and spleen, which functions of the marrow and of the lymphatic glands he further considered to remain constant throughout adult life, so that myelocytes and granular cells were never produced in the lymphatic apparatus, and *vice versa*, myeloid leukæmia was regarded as having its origin in disease of the bone-marrow, and lymphatic leukæmia in the lymphatic glands. Recent work, and especially the discovery of cases of lymphatic leukæmia with lesions limited to the marrow, has, however, shown that the latter distinction can no longer be regarded as a constant one, and perhaps for a time too exclusive attention in diagnosis had been paid to the characters of the blood, and to preconceived theories of the seat of origin of the leucocytes, and not enough to the actual changes in the organs. The tendency of late has been to attach less importance to the precise seat of the pathological changes, but a right understanding of the disease can, of course, only be obtained by careful correlation of all its features.

As you know, two forms of leukæmia can easily be distinguished by the striking differences in the hæmatological details, clinical signs, and pathological changes, which are present in typical cases of each, namely (1) myeloid, myelocytic leukæmia (myelæmia), and (2) lymphocytic, lymphoid, or lymphatic leukæmia (lymphæmia).

Either of these varieties may occur in an acute or chronic form. Chronic myelocytic leukæmia is more common than the chronic lymphoid variety, and we might, therefore, expect that the acute myelocytic form would also be more frequent than the acute lymphocytic, but this is not the case. Indeed, the recognition of acute myelocytic leukæmia has only been recently accomplished, and until lately the existence of such a form was denied by many authorities. The distinction of typical cases of the two varieties presents no difficulty, the blood-picture and the histological changes in the tissues being entirely different. In atypical cases, however, and especially in certain cases of the acute variety, the difficulty of distinction may be great, but it will be better to defer the consideration of this question until we come to deal more especially with acute leukæmia.

The following table, to which I have appended the corresponding blood changes, gives a summary of the pathologico-anatomical changes in myeloid and lymphatic leukæmia respectively, and is taken from an article by Fabian, Naegeli and Schatiloff.[2]

| Myeloid Leukæmia. | Lymphatic Leukæmia. |
|---|---|
| *Marrow.*—Hyperplasia of the tissue of the bone-marrow at the expense of the fatty marrow ; myeloid growth ; the ordinary loose cell-structure with abundant capillaries, and rich reticulum remains unaltered, as well as the formation of erythrocytes. | Growth of lymphoid tissue, resulting in a close lymphoid tissue, at first in nodules, then diffusely, in which remains of myeloid tissue are present, or have altogether disappeared. Capillary vessels and erythro-poietic areas scanty. |
| *Spleen.*—Collections of myeloid tissue start in the splenic pulp, spread through it, and cause a contraction or disappearance of the follicles, resulting in a "myeloid spleen." | Growth of lymphadenoid tissue ; increase in size of the follicles, and gradual replacement of splenic pulp by lymphoid tissue. |
| *Liver.*—Extensive myeloid growth, partly intra-capillary, by which the liver-cells are compressed, and may disappear, partly in intra-acinous tissue, perivascular, in form of streaks or bands, never rounded or like follicles ; occasionally slight extra-acinous growth. | Growth of lymphoid tissue, which is exclusively intra-acinous, and often very sharply defined. |
| *Lymph glands.*—A growth of myeloid tissue which at first leaves the follicles intact, and often shows only slight adventitious areas centrally, but finally the whole gland may undergo a myeloid transformation with disappearance of the follicles. | Growth of lymphoid tissue occurs, which in the end changes the gland into a structureless collection of lymphatic cells, in which the remains of the follicles can often scarcely be recognised. |

The authors could not satisfy themselves in any instance that a direct transformation of lymphoid tissue, including lymphatic follicles, into myeloid tissue occurred, or *vice versa.*

*Blood.*—The changes in the blood-picture can be best seen from the slides which I show. They are all too well known to render it necessary for me to describe them in detail. Briefly, the chief feature in myeloid leukæmia is the presence in great, often enormous numbers, of neutrophil myelocytes, of eosinophil myelocytes, and of mast cells ; the neutrophil myelocytes may vary in number from 10—80 per cent. of the leucocytes present. Normoblasts are present, generally in considerable, often in large numbers, and also megaloblasts. There are also present cells like the large lymphocytes of acute lymphatic leukæmia. The red cells show generally a moderate reduction in number, with a corresponding fall in hæmoglobin. In lymphoid leukæmia the chief characteristic is the great increase in the lymphocytes, or mononuclear non-granular cells. The total leucocyte count may vary from 30,000—550,000 to c.mm. (Pinkus), and of these the lymphocytes constitute from 90—99 %. Two forms of lymphoid leukæmia can be clinically distinguished according as the cells present consist of (1) small, or (2) large lymphocytes (using the term lymphocyte provisionally for the moment). A few myelocytes are generally seen. The erythrocytes may show a moderate decrease, but in acute cases the fall in the reds is often rapid and extreme, even to a number of less than 1,000,000 to c.mm. The hæmoglobin is often disproportionately reduced, so that the colour index is less than 1. In some cases there are not many normoblasts ; in others normoblasts are very numerous, with marked changes in their nuclei and megaloblasts are present. According to McCrae,[3] however in forty cases taken from the literature the number of red cells was low, and the colour index high in a large proportion of them, and in many cases there were many nucleated reds.

We may consider chronic myelocytic leukæmia first, as there is less difference of opinion about its nature and mode of origin. The origin of this form of leukæmia in the bone-marrow is now generally accepted, since the myelogenous origin of the myelocytes which characterise the blood-picture was established by Ehrlich. In some cases, however, the extent of the myeloid changes in the lymphoid tissues of the body make it possible that the marrow is not always the sole source: for instance, Fabian, Naegeli, and Schatiloff[4] look upon the spleen as the organ most constantly and almost invariably affected in all forms of leukæmia.

As to the nature of the change in the marrow, if as Ehrlich holds the myelocytes in the blood are identical with the myelocytes which are normally present in the marrow, and are the precursors of the polymorphonuclear leucocytes, then the change in the marrow must be looked upon as a kind of hyperplasia, and myelocytic leukæmia as a specific and essential disease of the marrow. The change in the marrow and the passing out from it of myelocytes into the blood is proved, but that the myelocytes found in the blood and organs are cells normal to the marrow ("unripe" cells) is not yet certain. Schur,[5] for instance, looks on the myelocytes of leukæmia as abnormal cells derived from a diseased marrow, and advances the well-known facts that in cases of myeloid leukæmia, in which an intercurrent infective disease occurs, myelocytosis may give way to an ordinary (polymorphonuclear) leucocytosis, and that in cases, *e.g.* of pneumonia, which are ordinarily accompanied by pronounced leucocytosis, the presence of myelocytes is altogether exceptional, so that stimulation of the marrow does not under ordinary conditions give rise to the passage of myelocytes into the blood, but quite regularly of polymorphonuclears. Against this, however, may be set the observations of a myelocytosis in certain anæmic conditions, and especially in septicæmia, in which in one case Naegeli found 20 per cent. of myelocytes.[6]

That the ordinary functions of the marrow can be performed in myelocytic leukæmia is shown by the occurrence of a normal leucocytosis in intercurrent infective disorders, and also by the absence as a rule of very profound anæmia. That it is undergoing some abnormal stimulation, and that cells, ordinarily confined to the marrow and normal to it, pass into the blood is also shown by the number of normoblasts and megaloblasts often present in the latter.

How do the myelocytes pass into the blood? Such passage of leucocytes into the blood is seen as a normal function of the marrow in an ordinary leucocytosis, and if we take the view that the myelocytes are precursors of the polymorphonuclears, these may be supposed to pass in the same way. Ehrlich states that both neutrophil and eosinophil myelocytes have the power of amœboid movement, and wander into the blood in myeloid leukæmia, looking on the process as an active leucocytosis. Such a leucocytosis might take place through chemiotaxis. Pappenheim, however, considers that the myelocytes are forced into the blood-stream by the pressure of the hypertrophied marrow enclosed in its unyielding bony envelope.

The origin of the myeloid tissue, containing myelocytes, in the spleen, liver, glands, and other organs, is a subject of greater difficulty. This process is peculiar to leukæmia, and has no exact pathological analogue in any other disease. Three views are held with regard to it: (1) That the cells originating in the marrow are carried by the blood-stream to the organs affected, lie up in them, and subsequently divide and proliferate; (2) that the myeloid growths in the organs are a development of a myeloid tissue which was previously latent; and (3) that there is a new formation of myeloid tissue in the affected organs, a myeloid metaplasia (Pappenheim).

It is reasonable to suppose that the myeloid formations in the organs—spleen, liver, glands, &c.—depend on the same cause that produces the change in the marrow, but also that in most cases the former are secondary in time to the latter.

As to (2) there is no evidence whatever of myeloid tissue lying latent in the spleen, glands, &c. But there is a modification of this view, namely that lymphoid tissue has the property under certain pathological conditions of undergoing a myeloid transformation. Recent work has rather tended to obliterate the sharp line of distinction until lately held to exist, chiefly on the basis of Ehrlich's views, between the functions of lymphoid and myeloid tissue, and to show that in pathological conditions lymphoid tissue may no longer produce only non-granular cells and lymphocytes and the marrow only granular cells, but that their functions may be interchanged.[7]

Ehrlich, Meyer and Heineke[8] agree in this view so far that the myeloid formation in the glands does not arise from the lymphocytes of the follicles, but consider that it comes out of other undifferentiated cells, which are similar to the lymphocyte-like cells of the marrow or myeloblasts. I may remark with regard to this, that if it were proved that the myelocytes of myeloid leukæmia were not the normal myelocytes of the marrow, but abnormal cells, they would reasonably be supposed to come from these lymphocyte-like marrow cells or myeloblasts, and in this way the myeloid formations of both marrow and other organs would be brought into line. In this connection it may be observed that Buckmaster[9] has shown that in a case at first regarded as lymphatic leukæmia the large lymphocyte-like cells by careful staining proved to be granular, and to really belong to the class of myelocytes.

Cabot[10] looks upon leucocytic hyperplasia as the essential feature of leukæmia, and thinks that it may begin in any part wherever hæmopoietic or lymphoid tissue exists. He postulates the essential unity of the hæmopoietic system, and that it is not sharply differentiated into myeloid and lymphoid elements; this underlying unity renders comprehensible the myeloid transformation of the glands in myeloid, and the lymphoid transformation of the marrow in lymphatic leukæmia. Reversion to a less specialised type of cell formation takes place, and results, if the marrow type prevails, in bringing the spleen and glands into line by a more or less widespread myeloid transformation; if the lymphoid type prevails, the marrow is brought into line with the lymph glands. He considers this explanation more in harmony with the facts than any theory of metastasis or transplantation of marrow cells or lymphocytes.

It may be pointed out, however, that the ascertained fact is that non-granular cells in the blood can under pathological conditions come from the marrow. That lymphoid tissue in glands, &c., manufactures granular cells and myelocytes is not so certain. The transplantation of lymphoid cells into the marrow is not in question, as it is known to contain lymphoid tissue, and according to Cabot himself the leucoblastic areas in it contain 15 to 20 per cent. of cells indistinguishable from large lymphocytes. On the other hand, whilst lymphoid tissue is normally widely distributed through the body, myeloid tissue is not found in the spleen, liver and lymph glands under normal conditions; if it were present, we should expect to find myelocytes at any rate occasionally in them. Further, myeloid leukæmia should then be common in children, for in them reversion to a less specialised type of the lymphatic apparatus should more easily occur, especially as there is reason to hold that the marrow in them is worked to its fullest capacity,[11] whereas we know that myeloid leukæmia in infancy and childhood is very rare Again, the myeloid changes in the lymphatic apparatus appear to be more of a secondary character in myeloid than the lymphoid change in the marrow in lymphatic leukæmia. Although, therefore, Cabot's view simplifies the problem of leukæmia, it does not seem to account for all the facts, for which, in the absence of exact knowledge with regard to the etiology of the disease, no one of the present theories gives a complete explanation. Indeed, until the essential cause or causes of leukæmia are known, we cannot expect to fully understand its nature, however extensive and minute our acquaintance with its morbid anatomy may be.

Passing on to lymphocytic, lymphoid or lymphatic leukæmia, there is great difference of opinion as to the nature and origin of the cells found in the blood in certain forms of it. Much stress in the differentiation of different forms of leukæmia has been laid on the morphological characters of cells. It is certain that caution is necessary in basing arguments as to the nature of diseases on minute differences in the histological structure of cells; if this is done, the characters of all parts of the cell, both nucleus and protoplasm, should be taken into consideration. Further, the technique of preparing and staining blood-films allows great variation in individual work, and it is quite possible to obtain divergent results from the same material, as in the case of Buckmaster above quoted.

The cells of the large lymphocyte type are often extremely fragile, easily damaged in preparing a film, and if the total leucocyte count is made by the method of using a weak acetic acid solution many may disappear.[12] In stained films, apart from technique, the recognition of a cell as belonging to one particular kind is sometimes a matter of opinion. Further, there is the difficulty of good differential staining of the leucocytes in sections of the tissues, though it is hardly necessary to say that no proper pathological diagnosis of leukæmia can be made on blood-films alone without a complete examination of the tissues. These difficulties in investigation and others of a similar kind probably account for some of the discrepant statements about the characters of the cells present in the different forms of leukæmia, and even, perhaps, sometimes for the description of new varieties of the disease.

In lymphatic leukæmia the acute form of the disease is frequent, and we are met at the outset with two questions: (1) What constitutes an acute case? and (2) does the character of the cells present in the blood and of the changes in the organs differ in acute and chronic cases?

The best answer to the first question is that acuteness should rest on the rapidity of the development of the blood-changes. Mere duration is an uncertain criterion. The onset of the disease is often difficult to fix precisely; and if the duration is taken as the criterion of acuteness, Sternberg suggests that it should not exceed four months.

In answer to the second question, many authors have regarded acute as synonymous with large-celled and chronic with small-celled lymphatic leukæmia. This distinction, although it may, perhaps, be taken in a rough way as true, no longer holds good in any exact sense. For example, Forbes and Langmead [13] bring forward four cases in which an acute course was accompanied by an increase of small lymphocytes in the blood, and one chronic case with large lymphocytes.

Cases in which the small lymphocyte is the predominating cell are not very common, and seem to form, at any rate from the blood-picture, a well-marked variety of the disease. The occurrence of intermediate varieties makes it impossible, however, to recognise any essential difference between the small and the large-celled forms. Lymphoid leukæmia differs in an important respect from myeloid, in that lymphoid tissue is generally recognised as widely distributed throughout the body; in most cases of the disease this tissue is very extensively affected, and hence this form of leukæmia constitutes a systematised disease or affection of one tissue-system throughout the body. The general age incidence is earlier in lymphoid than in myeloid leukæmia.

There are many conditions in which lymphoid tissue undergoes a rapid increase. In infancy and early life it shows a great activity, and diseases of the lymphatic apparatus are correspondingly common. To this activity of the lymphatic apparatus is also partly to be attributed the peculiar feature of the infant's blood, the high lymphocyte count.[14] As a result, lymphatic leukæmia in infancy takes mostly the acute form. Ewing[15] notes the occurrence of lymphocytosis in the secondary anæmia of children and in von Jaksch's anæmia. Pinkus[16] points out that the readiness with which a quiescent lymphoid tissue in any organ passes into a proliferating focus is seen in every inflammation in which an immense round-celled infiltration may arise from traces of lymphadenoid tissue, and further may throw an excess of cells into the lymph circulation. The rapid accumulation of cells which morphologically are identical with lymphocytes is well seen in some acute affections of the central nervous system, *e.g.* acute anterior polio-myelitis. He considers that the formation of lymphocytes is a normal function of every part of the body in response to local inflammatory irritation, and contrasts it with a polymorphonuclear-cell-infiltration or suppuration in which these cells, formed and stored up in the marrow, are rapidly carried by the blood-stream to the affected part.

These and other arguments at first sight lend much support to the old view that lymphocytic leukæmia is due to a widespread hyperplasia of the lymphatic apparatus, varying in degree and extent in different cases, the lymphoid tissue of the marrow being only subordinately affected, together with a peculiar blood state; and I believe this to be the true pathology of a certain number of cases of the chronic, and possibly also of some of the acute form, in which the predominating cell in the blood consists almost entirely of the easily distinguished small lymphocyte. The more chronic the case, as a rule, the more pronounced the enlargement of the glands. Thus Rosenfeld,[17] in three cases of chronic lymphocytic leukæmia, states that the primary seat of the disease was in the lymph-glands.

Recent work has, however, shown the importance of the part played by the bone-marrow in lymphocytic or lymphatic leukæmia, especially in its acute forms.

In cases of the disease recently reported, in which the condition of the bone-marrow has been investigated, it has been found to be altered—more or less in the manner given in the table above—and in some well-authenticated cases the morbid change has been entirely confined to the bone-marrow. This change may be limited in distribution, as in one very instructive case of Emerson's,[18] which shows the need of a thorough investigation, for in this the marrow of the vertebræ was alone affected, and that of the long bones was healthy. Holst[19] found streptococci in the marrow in three cases of acute lymphatic leukæmia, one of which he considers as distinctly due to a streptococcic infection of the marrow. Pappenheim regards lymphatic leukæmia as a lymph-adenoid hyperplasia having its seat in the bone-marrow; he and Wolff say that when the pathological process attacks the lymphatic apparatus only, the blood is unaffected, and the condition is not one of true leukæmia. Drysdale[20] states that the changes may be only in the bone-marrow, or part of it. Cabot[21] gives the following statement: (1) In most if not in all cases of lymphatic leukæmia there is a lymphoid transformation of the marrow; (2) that there is no reason to think that this is a metastasis secondary to the glands; (3) in a certain number of cases the glands and spleen are not enlarged, the changes being in the marrow and blood; and (4) in some cases the glandular enlargement appeared late in the disease. He does not take the above facts as showing the origin of all leukæmias in the marrow, but rather that the whole hæmatopoietic system is involved in every case of leukæmia, the changes sometimes preponderating in the marrow and spleen, sometimes in the glands. Kelly[22] makes the observation that the conception of lymphatic leukæmia as a disease of the marrow does not compel us to derive all the cells (in enormous numbers in many cases) from the marrow itself; in part they may come from the lymphatic glands. This view seems most in accordance with the facts, as the theory advanced by some writers, that the bone-marrow is the sole source of the disease, leaves unexplained the marked changes present in the glands and lymphatic apparatus generally in the majority of cases.

Allowing that recent observations point to the marrow as affected, the next question is as to the evidence that the marrow can produce lymphocytes, and also that the large mononuclear cells present in lymphocytic leukæmia are true lymphocytes.

The marrow certainly contains lymphadenoid tissue, and this was found enormously increased in many reported cases of lymphatic leukæmia.[1]

According to Hammar,[24] about the beginning of the fourth month of intra-uterine life an infiltration of mononuclear leucocytes begins to appear about the middle of the diaphysis, and extends through the whole bone, so as to give the marrow a lymphoid character. The myelocytes, which can be seen at the fifth month, appear to be developed from these cells. By the end of intra-uterine life the histological differentiation of the marrow would seem to be as complete as in adult life.

The experiments of Flexner[25] are important in this connection. He prepared two cytotoxic serums—lymphotoxin and myelotoxin. On injecting myelotoxin, the marrow responded with an increased production of granular leucocytes and myelocytes. On injecting lymphotoxin, there was an increase of lymphocytes and non-granular mononuclears in the marrow.

The marrow, therefore, not only contains lymphadenoid tissue, but can also produce non-granular mononuclear cells, although it is probably not concerned in the production of lymphocytes in health. The second part of the question is one about which there is most controversy. About the small lymphocytes in the less common forms of lymphatic leukæmia there is little doubt ; they correspond to the small lymphocytes normally present in the blood,

[1] We must note in passing, however, that Veszprémi,[23] in a careful investigation, including fibrin-staining methods, of a case of acute leukæmia, comes to the conclusion that a network of fibrin has been mistaken for lymphadenoid tissue in the marrow by authors who describe it as hypertrophied.

and may be taken to originate, like them, in the lymph-glands. But with regard to the large cells, which make up the blood-picture of the leucocytes in the majority of cases of the disease, it is more than doubtful whether they correspond at all to large lymphocytes in the ordinary sense, and whether they are normal cells of the blood.

Ehrlich's conception, which is apparently well founded for the normal adult life, that the marrow produces granular cells only and the lymphatic glands and spleen non-granular (lymphocytes), and that the two are not interchangeable in function, cannot be reasonably held to remain absolutely true for pathological states. It must be borne in mind that although no interchangeability of function between the marrow and the lymphatic apparatus may occur in health, yet in disease the conditions might be altered.

Remembering the necessity of caution in differentiating cells histogenetically solely by morphological characteristics and staining peculiarities, the following statements may be made as to the large non-granular mononuclear cells of lymphatic leukæmia.

These cells are mostly about three times the size of a red cell. They are delicate, and may smear or become froth-like in preparing the film. The nucleus is large, occupies about threequarters of the cell, sometimes stains badly, and gives an appearance of vacuoles. The protoplasm stains fairly, and in some shows a faint granulation. The cell is spherical, but its outline is often irregular or ragged.

Another cell is also met with in acute leukæmia, which is smaller than the above, and has a more densely-staining protoplasm and relatively larger nucleus.

These cells are now generally considered as abnormal to the blood. Such cells are, however, present in the normal marrow, but not in large numbers. Veszprémi[26] states that their number varies under various pathological conditions ; for instance, they are increased in septic and infectious diseases, and undergo an enormous increase in acute leukæmia. Many writers have suggested that they correspond to an undifferentiated marrow cell, antecedent to the stage of formation of the granular myelocyte. As we suppose that in the normal bone-marrow of the adult granular myelocytes and polymorphonuclears reproduce their own kind by division and self-multiplication, the appearance of these cells may be regarded as a reversion to an earlier fœtal stage in leucocyte formation. These cells would then correspond to the cells variously named by different observers, but whose characters shade into one another, as "indifferent lymphoid cell," Wolff; "lymphocyte," Pappenheim; "myeloblast," Naegeli; "stamm-zellen," Grawitz; and "lymphoid cell," Türk. Wolff[27] defines the cell as a lymphocyte of the bone-marrow, morphologically distinguished from the ordinary large lymphocyte, and capable of differentiation into various forms of leucocyte. This view of a common ancestor of the various leucocytes is, however, regarded by Sternberg[28] as one which, although of great probability, has no direct proof.

But leaving aside the question of a common ancestor for all leucocytes, we may suppose that in lymphatic leukæmia a mononuclear non-granular cell, antecedent in development to the myelocyte, develops in enormous numbers in the marrow, and passes into the blood. The affection of the marrow would, presumably, be more profound than in myeloid leukæmia, because it would be attacked at a phase earlier in the course of development of the generations of cells which result in the red-blood cell, and therefore the very profound anæmia which is found in so many cases of acute lymphatic leukæmia, and is so much more a feature of the disease than in myeloid leukæmia, would be explained.

The mode of dissemination of the cells in lymphatic leukæmia requires a word. So far as the cells are derived from the marrow, even though in an antecedent stage to the formation of myelocytes, they might make their way out in the same way as the latter. Wolff and others regard these cells (lymphocytes) as actively mobile, and as capable of amœboid movement, contrary to the older opinion; others think they can change their shape, but not their place. Probably where they lodge in the tissues they are capable of self-multiplication,[29] and it may be stated again that this conception of the important part played by the marrow in lymphatic leukæmia does not exclude the lymphatic apparatus generally from being also concerned.

With regard to the ætiology of acute lymphatic leukæmia, there is evidence that some cases may be due to a streptococcic infection. Holst's cases of streptococcic infection of the marrow have been mentioned. Forbes and Langmead obtained a streptococcus from the blood of four cases *post-mortem* and of one during life, although they are careful to point out that the organisms obtained *post-mortem* may have been due to a terminal infection. In many cases of this disease the rapidly fatal course—high fever, hemorrhages, and purpuric eruptions, together with the frequent presence of buccal and pharyngeal lesions—is strongly suggestive of a severe septic infection.

We are now in a position to consider acute myeloid or myelocytic leukæmia. The account just given of the share taken by the marrow in the production of acute lymphatic leukæmia brings it into close relation with acute myeloid leukæmia. In the former it is supposed that the affection of the marrow takes place at some stage antecedent to the development of the non-granular cells into myelocytes, *i.e.* at some preceding generation; in the latter it may be supposed to take place at a little later stage. It is easy to understand that the distinction between the two, especially in irregular or atypical cases, may be difficult or impossible. McWeeney[30] points out the impossibility of distinguishing by their morphology alone the undifferentiated (embryonic) cells of one tissue (myeloid) from those of another (lymphoid). The presence or absence of granules in the cells will be important; and this again depends to some extent on technique, one man will demonstrate them where another fails. Some cases described as acute lymphatic leukæmia in all probability really belong to acute myelocytic leukæmia.

Cases of acute myeloid leukæmia have now been recorded in which the changes in the blood and organs are sufficiently characteristic. Thus the blood contained neutrophil and eosinophil myelocytes, large and small mononuclears, small lymphocytes, polymorphonuclears, and a considerable increase in mast cells and eosinophils; a more mixed blood-picture than is seen in lymphatic, and giving the characteristics of myeloid, leukæmia. A myeloid transformation was found in the spleen, liver and glands.

Based on the views which I have discussed as to the nature of lymphatic leukæmia, two new forms or names have been suggested, (1) indifferent lymphoid cell leukæmia (Wolff), and (2) "myeloblasten" leukæmia. Wolff[31] postulates in blood-development (1) lymphocytes proper, (2) indifferent lymphoid cells (the cells we have been considering), which in their further course may develop into either large lymphocytes or myelocytes. Hence the "indifferent lymphoid-cell leukæmia" may develop into lymphoid or myeloid leukæmia. It seems to me that the decision in any particular case between "indifferent lymphoid cells" and "large lymphocytes" would be extremely difficult—understanding by the latter term the large mononuclear cell of lymphatic leukæmia—and the distinction of the corresponding leukæmic states very obscure.

Fabian, Naegeli, and Schatiloff[32] propose the term "myeloblasten" leukæmia, using Naegeli's term myeloblast as given above. They give four cases reported by Ziegler and Jochmann,[33] two cases of acute myelocytic leukæmia by Hirschfield,[34] and also a case under their own observation, in which the liver showed an enormous intracapillary growth of cells compressing the acini, the spleen a typical myeloid transformation, and there were also myeloid collections in the not enlarged lymphatic glands. They propose the following criteria for the differential hæmatological diagnosis: (1) The reactions to two special stains; (2) the presence of numbers of cells of all intermediate stages between myeloblasts and myelocytes; (3) a large number, not rapidly diminishing in the course of the case, of other myeloid elements, namely ordinary eosinophil and neutrophil myelocytes; (4) the presence in many cases of large nucleated red-blood cells; and (5) the demonstration of giant cells belonging to the marrow.

On the whole, it seems to me that there is hardly sufficient ground for the separation of either of these as separate varieties of acute leukæmia, nor is it advisable at the present stage of investigations into leukæmia to add to the already confused nomenclature new names for forms which, after all, are ultimately based on theoretical considerations as to the nature and ontogeny of the cells present. Lastly, the question of the occurrence of mixed forms of leukæmia is still not satisfactorily settled.

I now pass on to briefly consider the relation of leukæmia to two diseases of the bone-marrow—chloroma and myeloma; and to one of the glands—lympho-sarcoma.

In chloroma there are growths, of a peculiar bright green colour, most commonly primarily situated in the periosteum of the frontal and temporal bones, very often in or around the orbit. The growths may, however, begin in the vertebræ. The tumours also affect other parts of the skeleton, *e.g.* sternum, ribs and long-bones. Treadgold[35] considers that the growths begin definitely in the red marrow, which at puberty is restricted to the bones of the skull, vertebræ, ribs, sternum and epiphyses of the long-bones.

In other cases the chief changes are in the lymphatic apparatus, the bones being only slightly or not at all affected,[36] whilst the lymphatic tissue is very extensively involved, especially the lymphatic glands, in which the peculiar grass-green tumours are also found. The spleen is enlarged in all cases.

Other atypical cases are described which present all the typical features of chloroma without the green colour. The blood shows a marked leucocytosis, more or less pronounced in individual cases, in some enormous, to 800,000 cells to c.mm., and the increase consists in large mononuclear cells, from three to five times the size of a red-blood corpuscle, and which, according to Treadgold, contain more protoplasm than the similar cells of acute leukæmia. He describes two types of these cells. There is often profound anæmia. In the marrow, spleen, lymphatic glands and lymphatic apparatus generally the chief histological change is the presence of enormous numbers of similar mononuclear cells. Some of these cells probably contain neutrophil granules, and in several cases eosinophil cells—like eosinophil myelocytes—have been demonstrated in the tissues, but not in the blood. An infiltration of the liver and kidneys by mononuclears is also found.

Sternberg[37] makes a division into (1) lymphoid, (2) myeloid chloroma; in (1) there is an atypical growth of lymphoid, and in (2) of myeloid tissue elements. The first form he identifies with a disease called by him leuko-sarcomatosis. In leuko-sarcomatosis there is an increase of large mononuclear cells in the blood, at first relative, later absolute and very great. Similar cells are present in large numbers in the spleen, lymph-glands, thymus, tonsils, lymphoid tissue generally, and in the bone-marrow. The spleen and glands are enlarged, and the growths in the latter may spread beyond the glands to invade the periphery. The changes, in fact, correspond to those of chloroma, except that the green colour and also the extensive sub-periosteal growths of the latter are absent. In fact, the cases correspond to those forms of chloroma in which the stress of the disease falls on the lymphatic apparatus. These cases again present close resemblances to some forms of lymphatic leukæmia. The distinction is made by Sternberg partly on the basis of the blood changes and character of the large mononuclears, and partly on the tendency of the growths in the glands, &c., to invade the neighbouring tissues.

Looking upon lymphatic leukæmia as a hyperplasia of lymphatic tissue, he proposes the name "leuko-sarcomatosis" for the above condition, in which there is an invading atypical growth with heterotopous growths plus the leukæmic blood picture. The objections to this view are that, as has been said above, it is at least doubtful whether the large mononuclears of lymphatic leukæmia are normal to the blood, and that no distinction as to essential difference of kind can be based on mere size of the cells, varying as they do so much in individual cases; that if the cells of lymphatic leukæmia are not normal cells the infiltrations of the organs are in a sense heterotopous; and further, in some cases described, at any rate, as lymphatic leukæmia[38] there has been infiltration of the fat around the enlarged glands, tonsils, thymus, &c., both in the large and small-celled forms. I do not wish, however, to emphasise this last point further than to indicate that there is great difficulty in differentiating atypical cases of leukæmia from some allied conditions, and that these cases, called by Sternberg leuko-sarcomatosis, afford a transition between lympho-sarcoma and lymphatic leukæmia.

To return to chloroma, Treadgold[39] has shown the striking similarity between acute lymphatic leukæmia and chloroma in incidence of age and sex, and also in symptomatology. He also makes the interesting suggestion that the greater richness in protoplasm of the mononuclear cell in chloroma may account for the greater activity of the growths in invading the surrounding tissues.

Brief reference may be made in this connection to myeloma, a disease characterised by a systemic affection of the lymphatic-hæmatopoietic apparatus, associated with local tumour-like formations in bone, hyperplastic in nature, that is to say, showing no histological elements which are not normally present in bone-marrow.[40] The pathognomonic symptom of Bence-Jones's albuminuria is well known. Myeloma has nothing to do with other tumours of the bone-marrow, but is closely allied to the leukæmias. There is, however, no blood change beyond secondary anæmia. A slight leucocytosis has been described in some cases, and in others myelocytes have been found in the spleen, liver and glands. Lubarsch recognises four types of cell present in the tumours:—(1) Myeloma. The cell a true myelocyte with or without granules. (2) Lymphocytoma. The cell a lymphocyte, from lymphoid tissue of marrow. (3) Erythroblastoma. The cell belongs to type of megaloblasts. (4) Leukocytoma. The cell large, non-granular mononuclear. He accordingly divides myeloma into myelocytoma, lymphocytoma, and erythroblastoma.

Ribbert[41] assigns a special position to myeloma, in that the tumours contain as characteristic constituent parts the forerunners of red cells or erythroblasts. In any case, there seems to be in myeloma a local tumour of the marrow, the prevailing cell varying in different cases, but being always a normal constituent of the marrow.

The last disease of the lymphatic apparatus of which I wish to speak is lympho-sarcoma, which, as a new growth, shows some peculiar features which have always made it rather a puzzle. Lympho-sarcoma is a local growth which does not affect the lymphatic apparatus in a general manner ; it has a local malignancy, invading surrounding non-lymphatic structures. The spleen and the bone-marrow are not often affected, or if affected, only in a local and circumscribed manner: the growths may undergo necrosis. In all these points, and in the absence of affection of the blood, lympho-sarcoma differs from lymphatic leukæmia, whilst it differs from ordinary sarcoma of the lymph-glands by starting locally in a gland, or in lymphoid tissue, *e.g.* of the fauces, by forming one growth and spreading along the lymphatic tissue paths, whilst sarcoma spreads irregularly and often by multiple growths in different parts. Lympho-sarcoma, again, is distinguished from lymphatic leukæmia as an atypical growth of lymphadenoid tissue, destroying and in no way resembling the normal lymphoid tissue. Observations on the blood are not sufficient at present, in most cases there appears to be no leucocytosis, but some have found a moderate leucocytosis, others a lymphocytosis. The causes of lympho-sarcomatosis are varied: in some cases there is a bacterial infection, in others irritation of the bronchial or mediastinal glands, as in stone-cutters, in anthracosis, &c. Hence Paltauf supposes a constitutional predisposition to the disease.

It is thus seen that, as Cabot[42] points out, there are all transitions from ordinary sarcoma through Sternberg's leuco-sarcomatosis and through myeloma to leukæmia of the ordinary type, and we must now consider the relationship of leukæmias to such growths. Some authors have considered leukæmia as a kind of sarcoma of the blood or blood-forming organs. Banti looks upon lymphatic leukæmia as a sarcomatosis, and on the infiltrations of the liver, &c., as hæmatogenous metastases. Wolff regards his "indifferent lymphoid-cell" leukæmia as a kind of tumour formation. Türk differentiates (1) small-celled leukæmia, (2) acute lymphomatoses, and (3) lympho-sarcoma, but groups them together, as he considers that they may all interchange with one another. Treadgold regards acute lymphatic leukæmia as a malignant tumour arising in the marrow of bone, chloroma as a tumour of myeloblasts of the red marrow, and acute myeloid leukæmia as of similar nature.

Forbes and Langmead pertinently remark, with regard to Treadgold's view of acute leukæmia, that malignant disease running such a very acute course as some of these cases do is unknown ; in fact, clinically, they much more nearly approximate to acute septic infections.

Some of the reasons which have been advanced for the tumour theory of leukæmia are : (1) That the cells found are atypical. In answer to this, both myelocytes and non-granular mononuclears are found in normal marrow, and myelocytes are also present in the blood in some anæmias, and occasionally in severe infections. (2) That infiltration of the capsule of glands, and of the periosteum of the bones occurs in certain cases of lymphatic leukæmia, not only in large-celled but in some small-celled and chronic cases. Borst and Pappenheim,[43] however, state, and in this are supported by Zeigler, that the microscopic appearance of such invasions is not decisive ; essentially there is only an inflammatory (granulation) tissue. (3) That the leukæmic infiltrations in the organs are to be looked upon as metastases. There is, however, little resemblance to tumour-metastasis in the infiltrations of myeloid or lymphatic leukæmia, the microscopic appearances, *e.g.* in the liver, spleen or kidney, are not suggestive of new growth, and these infiltrations do not excite inflammatory reaction in the tissues they invade. Also against the tumour theory, other observers have pointed out that the irregular mitoses which are found in the cells of malignant growths are not seen in those of leukæmic infiltrations : that there are no blood channels formed by the new cells such as are formed in sarcoma by sarcoma cells : that in lymphatic leukæmia, owing to the presence everywhere in the body of lymphoid tissue, it is difficult to regard the leukæmic growths as strictly heterotopous, no sharp line can be drawn between such cases and others in which the growths are truly heterotopous, and even in the latter case Zeigler considers that they are hardly of sufficient importance to assign leukæmia to the tumours. It must be admitted, however, that a decisive answer to the question of the relation of leukæmia to sarcoma cannot be given with our present knowledge, and that at present it is extremely difficult to draw the line at where lymphatic hyperplasia ends and tumour begins ; but the very fact of the indefinite number of gradations which are found between the cases at the two ends of the scale affords an additional argument against regarding leukæmia as a sarcoma.

In conclusion, I have purposely not used the term pseudo-leukæmia, as the name has been used in so many senses by different writers, that its employment almost inevitably leads to confusion. There are cases, however, in which leukæmic changes are found in the organs, but in which there is no affection of the blood—the so-called aleukæmic stage of leukæmia; and if, as has been proposed by Sternberg, the name pseudo-leukæmia was confined to these cases, it would have a definite signification.

### REFERENCES.

[1] **Buckmaster,** *Morphology of Normal and Pathological Blood,* 1906.
[2] "Beiträge z. Kenntniss d. Leukämie," *Arch. f. path. Anat.*, 1907, cxc. 474.
[3] *Brit. M. J.*, 1905, i. 404.
[4] *Loc. cit.*
[5] Quoted by Sternberg, "Primärenkrankungen des lymphat. u. häma-topoetischen apparatus," *Ergebn. d. allg. Path. u. path. Anat.* (Lubarsch-Ostertag), 1903, ii. 434.
[6] *See* **Cabot,** *A System of Medicine,* Edited by Osler and McCrae. Vol. iv., 1908.
[7] **Hutchison,** Goulstonian Lectures. "Some Disorders of the Blood and Blood-forming Organs in Early Life," *Lancet,* 1904, i. 1253 *et seq.*
[8] *Deutsches Arch. f. Klin. Med.*, 1907, lxxxviii.
[9] *Op. cit.*
[10] *A System of Medicine.* Edited by Osler and McCrae. Vol. iv., 1908.
[11] **Hutchison,** *loc. cit.*
[12] **Emerson,** *Johns Hopkins Hosp. Bull.*, 1907, xviii. 71.
[13] "Fatal Lymphocythæmia in Early Life," *Proc. Roy. Soc. Med.* (*Clin. Sect.*), 1908, i. 129.
[14] **Hutchison,** *loc. cit.*
[15] "Clinical Pathology of the Blood," 1901.
[16] **Nothnagel,** *Encyclopedia of Practical Medicine.* "Diseases of the Blood," 1905.
[17] *Arch. f. klin. Med.*, xliii. 1 and 2, quoted by Pinkus.
[18] *Johns Hopkins Hosp. Bull.*, 1907, xviii. 71.
[19] Quoted by Emerson, *loc. cit.*
[20] *Loc. cit.*
[21] *Quart. J. Med.*, 1907, i. 89.
[22] *Tr. Ass. Am. Physicians,* 1903, xviii. 481.
[23] Beiträge z. Histologie der sog. "akuten Leukämie," *Arch. f. path. Anat.*, 1906, clxxxiv. 220.
[24] Quoted by Hutchison, *loc. cit.*, p. 1255.
[25] Quoted by Treadgold, "Chloroma and Acute Lymphatic Leukæmia," *Quart. J. Med.*, 1907, i. 263.
[26] *Loc. cit.*
[27] Quoted by Ehrlich and Lazarus, Nothnagel's *Encyclopedia.*
[28] *Loc. cit.*
[29] **Veszprémi,** *loc. cit.*
[30] *Brit. M. J.*, 1905, i. 404.
[31] Quoted by Sternberg, *loc. cit.*

[32] *Loc. cit.*
[33] *Deutsche med. Wchnschr.*, 1905, nr. 30.
[34] *Berl. klin. Wchnschr.*, 1905, nr. 3, and 1907, xliv. 772.
[35] "Chloroma and Acute Lymphatic Leukæmia," *Quart. J. Med.*, 1907, i. 263.
[36] **Sternberg,** *loc. cit.*
[37] *Loc. cit.*
[38] **Fabian, Naegeli, and Schatiloff,** *loc. cit.*
[39] *Loc. cit.*, p. 261.
[40] **V. Lubarsch,** "Zur Myelomfrage," *Arch. f. path. Anat.*, 1906, clxxxiv. 214.
[41] Quoted by Sternberg, *loc. cit.*
[42] *Loc. cit.*
[43] Quoted by Fabian, Naegeli, and Schatiloff, *loc. cit.*

